# Mouse maternal odontogenic infection with *Porphyromonas gingivalis* induces cognitive decline in offspring

**DOI:** 10.3389/fped.2023.1203894

**Published:** 2023-08-11

**Authors:** Eri Ishida, Hisako Furusho, Ting-Yi Renn, Fumie Shiba, Hung-Ming Chang, Hiroshi Oue, Ryuji Terayama, Yukio Ago, Kazuhiro Tsuga, Mutsumi Miyauchi

**Affiliations:** ^1^ Department of Advanced Prosthodontics, Graduate School of Biomedical and Health Sciences, Hiroshima University, Hiroshima, Japan; ^2^ Department of Oral and Maxillofacial Pathobiology, Graduate School of Biomedical and Health Sciences, Hiroshima University, Hiroshima, Japan; ^3^ Department of Anatomy and Cell Biology, School of Medicine, College of Medicine, Taipei Medical University, Taipei, Taiwan; ^4^ Department of Maxillofacial Anatomy and Neuroscience, Graduate School of Biomedical and Health Sciences, Hiroshima University, Hiroshima, Japan; ^5^ Department of Cellular and Molecular Pharmacology, Graduate School of Biomedical and Health Sciences, Hiroshima University, Hiroshima, Japan

**Keywords:** maternal exposure, microglia, astrocyte, nervous system disease, periodontitis, *Porphyromonas gingivalis*

## Abstract

**Introduction:**

*Porphyromonas gingivalis* (*P. gingivalis*), a major periodontal pathogen, causes intrauterine infection/inflammation. Offspring exposed to intrauterine infection/inflammation have an increased risk of neurological disorders, regardless of gestational age. However, the relationship between maternal periodontitis and offspring functional/histological changes in the brain has not yet been elucidated.

**Methods:**

In this study, we used a gestational mouse model to investigate the effects of maternal odontogenic infection of *P. gingivalis* on offspring behavior and brain tissue.

**Results:**

The step-through passive avoidance test showed that the latency of the acquisition trial was significantly shorter in the *P. gingivalis* group (*p* < 0.05), but no difference in spontaneous motor/exploratory parameters by open-field test. *P. gingivalis* was diffusely distributed throughout the brain, especially in the hippocampus. In the hippocampus and amygdala, the numbers of neuron cells and cyclic adenosine monophosphate response element binding protein-positive cells were significantly reduced (*p *< 0.05), whereas the number of ionized calcium binding adapter protein 1-positive microglia was significantly increased (*p *< 0.05). In the hippocampus, the number of glial fibrillary acidic protein-positive astrocytes was also significantly increased (*p *< 0.05).

**Discussion:**

The offspring of *P. gingivalis*-infected mothers have reduced cognitive function. Neurodegeneration/neuroinflammation in the hippocampus and amygdala may be caused by *P. gingivalis* infection, which is maternally transmitted. The importance of eliminating maternal *P. gingivalis*-odontogenic infection before or during gestation in maintenance healthy brain function in offspring should be addressed in near future.

## 1. Introduction

Periodontitis is complex, multifactorial, polymicrobial infection and is most common infectious disease in the world. *Porphyromonas gingivalis* (*P. gingivalis*), a Gram-negative anaerobic bacterium is well accepted as the keystone periodontal pathogen (1). *P. gingivalis* is associated with several systemic diseases (2) including cardiac (3), respiratory (4, 5), and brain diseases (6, 7). *P. gingivalis* has harmful effects on the liver (8), coronary arteries (9), and placenta (10) through hematogenous translocation, inducing chronic systemic inflammation and/or changes in enteric bacterial flora (11). Periodontitis is prevalent in approximately 40% of pregnant women (12). A case-control study (13) showed that maternal periodontitis increased the risk of premature birth/low birth weight by 7.9-fold. *P. gingivalis* has been detected in the amniotic fluid of pregnant women at risk of premature delivery (14), and the placenta of women with chorioamnionitis (10), suggesting that *P. gingivalis* is the causative bacterium of intrauterine infection/inflammation.

Intrauterine infection/inflammation is a major cause of spontaneous preterm birth. Fetuses exposed to intrauterine infection/inflammation have a 2- to 5-fold increased risk of developing neurological disorders, regardless of gestational age (15). However, few studies have examined the relationship between maternal periodontitis and cranial neuropathy in children. Further, these studies have used an acute inflammation model induced by intrauterine injection of lipopolysaccharide (16, 17) or a *P. gingivalis*-induced systemic inflammation model (18). Limited studies have clarified the relationship between chronic inflammation in periodontitis and brain damage using animal models. we previously showed that *P. gingivalis* had reached the placenta and induced preterm birth by increasing inflammatory mediators (TNF-α, COX-2, and galectin-3) in a pregnant mouse model (19, 20).

*P. gingivalis* and its virulence factors have been detected in the brains of patients with Alzheimer's disease (21, 22). Animal and epidemiological studies also support an association between periodontitis and cognitive impairment (23, 24).

Here, we hypothesized that in pregnant mouse model with periodontitis, *P. gingivalis* migrates via the placenta to the brain of offspring, affecting cognition and learning through neuroinflammation. However, the mechanism and potential of *P. gingivalis* to induce brain damage in children remain unclear. The hippocampal–amygdala circuit is responsible for generating fear, emotional, memory, and defense responses. The hippocampus plays an important role in cognitive functions such as memory formation and consolidation (25). The amygdala contributes to the detection and avoidance of threats, while the hippocampus is involved in learning and contextual memory. The interaction between these two regions is critical for fear memory formation and avoidance behavior. Glial responses in the vicinity of amyloid plaques in Alzheimer's disease also correlate with the degree of cognitive impairment.

Using *P. gingivalis*-odontogenic infection mouse pregnant model, we aimed to clarify the effects of maternal odontogenic infection of *P. gingivalis* on glial cells in hippocampus and amygdala and on cognitive function of offspring.

## 2. Methods

### 2.1. Study design

**Figure 1:**
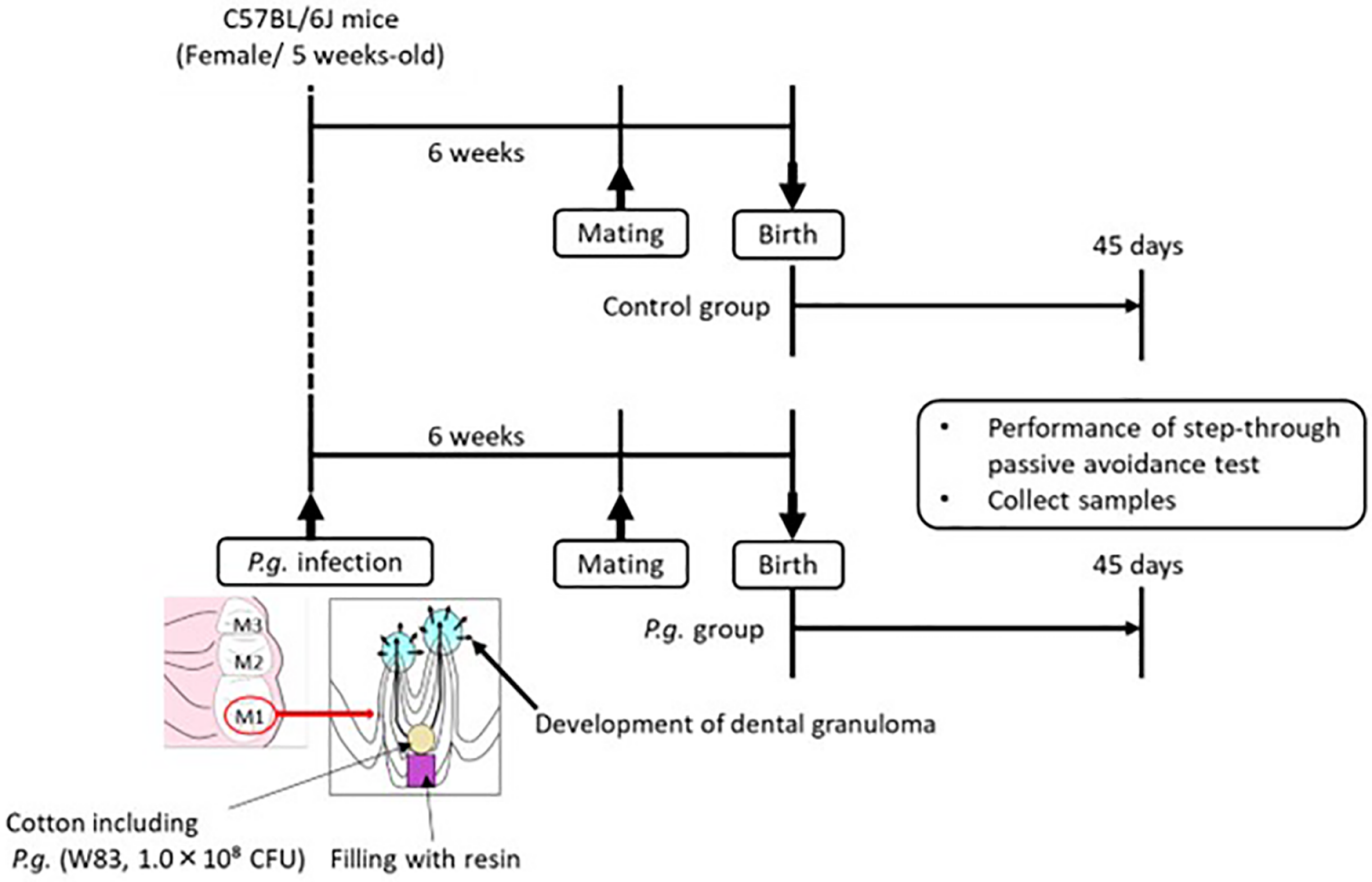
Five-week-old wild type female mice are infected with *P. gingivalis*, and mating is commenced 6 weeks after *P. gingivalis* infection. Passive avoidance test (PAT) of the offspring is performed at 45 days after birth, and brain tissue is collected after PAT.

Animal experiments were conducted in accordance with the “Guidelines for the Care and Use of Laboratory Animals” established by Hiroshima University. All experimental procedures involving animals were approved by the Ethics Committee for Animal Experimentation of Hiroshima University (approval number: A21–41) and were performed in accordance with the Animal Research: Reporting of *In Vivo* Experiments Guidelines and the American Veterinary Medical Association Guidelines on Euthanasia. The experimental protocol is shown in Figure 1.

### 2.2. Bacterial culture and count

*Porphyromonas gingivalis* W83 strain (Nissui Pharmaceutical Co. Ltd.) was seeded on sheep blood agar, cultured under anaerobic conditions in AnaeroPack (Mitsubishi Gas Chemical Co. Inc.) for 4 days at 37°C, and resuspended in phosphate-buffered saline (PBS). The number of bacteria was determined by measuring the optical density at 660 nm using a spectrophotometer.

### 2.3. Mouse model development

In a specific pathogen-free facility, 15 5-week-old female C57BL/6J mice (Charles River Japan Inc.) were exposed to a 12-h light/dark cycle with *ad libitum* access to autoclaved food and water. The mice were randomly divided into two groups: with or without odontogenic infection of *P. gingivalis*. Seven mice were infected with *P. gingivalis* W83 strain, as previously described (26). The roof of the pulp chamber of the bilateral maxillary first molars was removed. A small cotton swab containing 10^8^ colony-forming units of *P. gingivalis* was inserted into the pulp chamber and sealed with resin (Clearfil Majesty ES-2; Kuraray Noritake Dental Inc.). Mating was initiated 6 weeks after *P. gingivalis* infection, when periapical periodontitis was established. The offspring of *P. gingivalis*-infected mice (21 male, 27 female) were included in the *P. gingivalis* group; the offspring of uninfected mice (32 male, 30 female) were included in the control group.

Preterm infants are more susceptible to brain dysfunction and diseases due to brain immaturity (27) To exclude the effects of brain immaturity and to clarify the effect of intrauterine infection with *P. gingivalis*, a gestational age of >18.5 days was used as the cutoff for inclusion in the *P. gingivalis* group. The mean gestational age of offspring in the *P. gingivalis* and control groups was 19.5 and 20.0 days, respectively. The number of offspring per litter is defined as 4–9 to avoid variation in the size of body. There was no significant difference in litter size used between *P. gingivalis* and control groups (Supplementary Figure S1A). Experiments were performed at 45 days of age. At 45 days of age, there is no difference in body weight by the litter size of both groups (Supplementary Figure S1B).

### 2.4. Step-through passive avoidance test

Learning and memory functions were assessed by a step-through passive avoidance test. The Passive Avoidance System (Muromachi Kikai Co. Ltd.) consisted of two compartments separated by a guillotine door: a light compartment (90 × 115 × 150 mm) and a dark compartment (140 × 175 × 150 mm). For the acquisition trial, mice were placed in the light compartment, and the door between the two compartments was opened 15 s later. When mice entered the dark compartment, the door automatically closed and an electric shock (3 mA for 3 s) was delivered. The latency to enter the dark compartment was recorded. At 24 h after the acquisition trial, a retention trial was conducted for 300 s. The mice were again placed in the light compartment, and the latency to enter the dark compartment was recorded without electrical stimulation.

### 2.5. Open-field test

The open-field test was used to evaluate spontaneous motor/exploratory activity in mice. The apparatus consisted of a chamber (40 cm length, 40 cm width, 30 cm height). Each mouse was gently placed in the center area and was left free to explore the unfamiliar open field for 5 min. The time spent in the central and corner area, and total distances traveled were recorded and calculated by ANY-maze (Muromachi Kikai Co. Ltd.).

### 2.6. Tissue collection and neuron quantification

The right hemisphere of the brain was fixed in PLP solution (4.0% paraformaldehyde, 1.2% lysine hydrochloride, 0.2% sodium m-periodate) for 2 days at 4°C and embedded in paraffin.

Coronal sections (4.5 µm-thick; approximately –1.8 mm from Bregma) were Nissl stained to determine morphological changes and the number of neurons. The number of neurons with distinct nuclei, cytoplasm, and borders in the CA1 (100 × 300 µm) and CA3 (200 × 200 µm) regions of the hippocampus and basal (200 × 400 µm) and lateral (base: 300 × height: 500 µm, triangle) amygdala was counted in a blinded manner under a microscope, as previously described (28, 29).

### 2.7. Immunohistochemistry

Immunohistochemical staining for glial fibrillary acidic protein (GFAP) (an astrocytic marker), ionized calcium binding adaptor protein 1 (Iba1) (a microglial marker), cyclic adenosine monophosphate response element binding protein (CREB), and *P. gingivalis* was performed on paraffin-embedded tissue using the Envision Peroxidase System (Agilent Technologies Inc.) or Histofine Simple Stain MAX-PO Kit (Nichirei Bioscience Inc.). Sections were deparaffinized, and antigen retrieval was performed in citrate buffer (pH 6.0) for 1 h at ≥90°C (GFAP and Iba1). The sections were immersed in 0.3% hydrogen peroxide (Sigma-Aldrich) in methanol (Junsei Chemical Co. Ltd.) for 1 h to eliminate endogenous peroxidase activity. Protein Block (Agilent Technologies Inc.) was applied for 10 min at room temperature to eliminate non-specific binding.

The sections were then incubated with primary anti-GFAP (1:3,000) (ab7260; Abcam), anti-Iba1 (1:500) (ab5076; Abcam), anti-CREB (1:2,000) (#9197; Cell Signaling Technology), or anti-*P. gingivalis* whole rabbit polyclonal antibodies (1:500) (Prof. Kazuyuki Ishihara, Tokyo Dental College) overnight at 4°C and washed with PBS. For GFAP and Iba1, sections were incubated for 30 min with horseradish peroxidase-labeled polymer-conjugated rabbit immunoglobulin G (IgG) or Histofine Simple Stain MAX-PO goat IgG secondary antibodies. For CREB, sections were incubated for 60 min with horseradish peroxidase-labeled polymer-conjugated rabbit IgG secondary antibodies. For *P. gingivalis*, sections were incubated with goat serum for 10 min at room temperature followed by horseradish peroxidase-labeled polymer-conjugated rabbit IgG secondary antibodies for 30 min. Sections were counterstained with hematoxylin, immersed in graded alcohol and xylene, mounted, and visualized using DAB (Dako) or HistoGreen Substrate Kit for Peroxidase (Eurobio Ingen).

For astrocytes, microphotographs of CA1 and CA3 were taken at ×100 magnification, and the number of GFAP-positive cells was counted. Soma size, defined as the cross-sectional area of an outline around the GFAP-positive soma, was measured in two ranges per sample for each region [100 × 100 µm (*n* = 10)]. The average soma size was calculated. For microglia, microphotographs of CA1, CA3, and the amygdala (100 × 100 µm) were taken in two ranges per sample. The number of Iba1-positive cells in each region (>50 pixels included in the diameter of the soma) was counted and averaged. In the amygdala and cortex, “fractal dimension” (D values) was also analyzed. All images were captured on the same day by the same investigator to maintain the uniform settings adjusted at the beginning of capturing. The general approach to quantitative image analysis was similar to that reported previously (24, 25). A computer-based image analysis system and Image-Pro Plus Software (Media Cybernetics) were used to quantify morphometric alterations in positively labeled cells. The number and size of GFAP-labeled astrocytes and Iba1-labeled microglia were densitometrically determined. To quantify morphological changes in microglia (ranging from simple rounded to complex branched), D values were used, with higher D values indicating greater complexity (30). The mathematical approach to define D can be described as follows: where D as the exponent to which scale (*ε*) is raised to get the number of identical parts to itself [N*ε*; Equation (1)]. Therefore, D can be calculated from the ratio of lnN*ε* to ln*ε* [Equation (2)] (30).

(1) $$N\varepsilon =\varepsilon^{D}$$

(2) D=ln⁡Nε/ln⁡ε

The number of CREB-positive cells was counted in CA1(80 × 160 µm), CA3(120 × 120 µm), and the basal (200 × 400 µm) and lateral (base: 300 × height: 500 µm, triangle) amygdala.

### 2.8. Western blotting

GFAP and β-actin expression was examined by Western blotting. The hippocampus was minced into 2 mm squares and homogenized. Proteins were extracted using Tissue Extraction Reagent I (Thermo Fisher Scientific), Protease Inhibitor Cocktail (Sigma-Aldrich), and Bradford reagent (APRO Science). Protein concentrations were measured by absorbance using an iMark Microplate Reader (Bio-Rad). Loading buffer was added to the diluent containing 25 µg of protein and heated for 5 min at 95°C. Protein lysates were subjected to 10% polyacrylamide gel electrophoresis and transferred to polyvinylidene difluoride membranes. The immunocomplex was detected using an ECL Western Blotting Detection System (Amersham Biosciences). Primary anti-GFAP (1:10,000) (ab7260; Abcam) and anti-β-actin (1:5,000) (AC-74; Sigma-Aldrich) antibodies were used. Data were quantified using ImageJ (Wayne Rasband, NIH USA).

### 2.9. Statistical analysis

All data are expressed as the mean ± standard error of the mean (SEM). For the passive avoidance test, analyses were made using three-way analysis of variance (ANOVA) with *P. gingivalis* infection and sex as the intersubject factors and repeated measures with acquisition/retention trial as the intrasubject factor, followed by the Tukey–Kramer *post-hoc* test. For the open-field test, analyses were made using two-way ANOVA with *P. gingivalis* infection and sex as the intersubject factors, followed by the Tukey–Kramer *post-hoc* test.

For other data, the homogeneity of variance was checked by the *F*-test after performing Shapiro-Wilk's test. If equal variances were found, the Student *t*-test was used, and if not, the Welch *t*-test was used. For nonnormal distributed datasets, the Mann–Whitney *U* test was done. A value of *p *< 0.05 was indicative of statistical significance. All statistical analyses were performed using BellCurve for Excel (Social Survey Research Information Co. Ltd., Tokyo, Japan).

## 3. Results

### 3.1. Maternal *P. gingivalis* infection induced cognitive decline in male offspring

**Figure 2:**
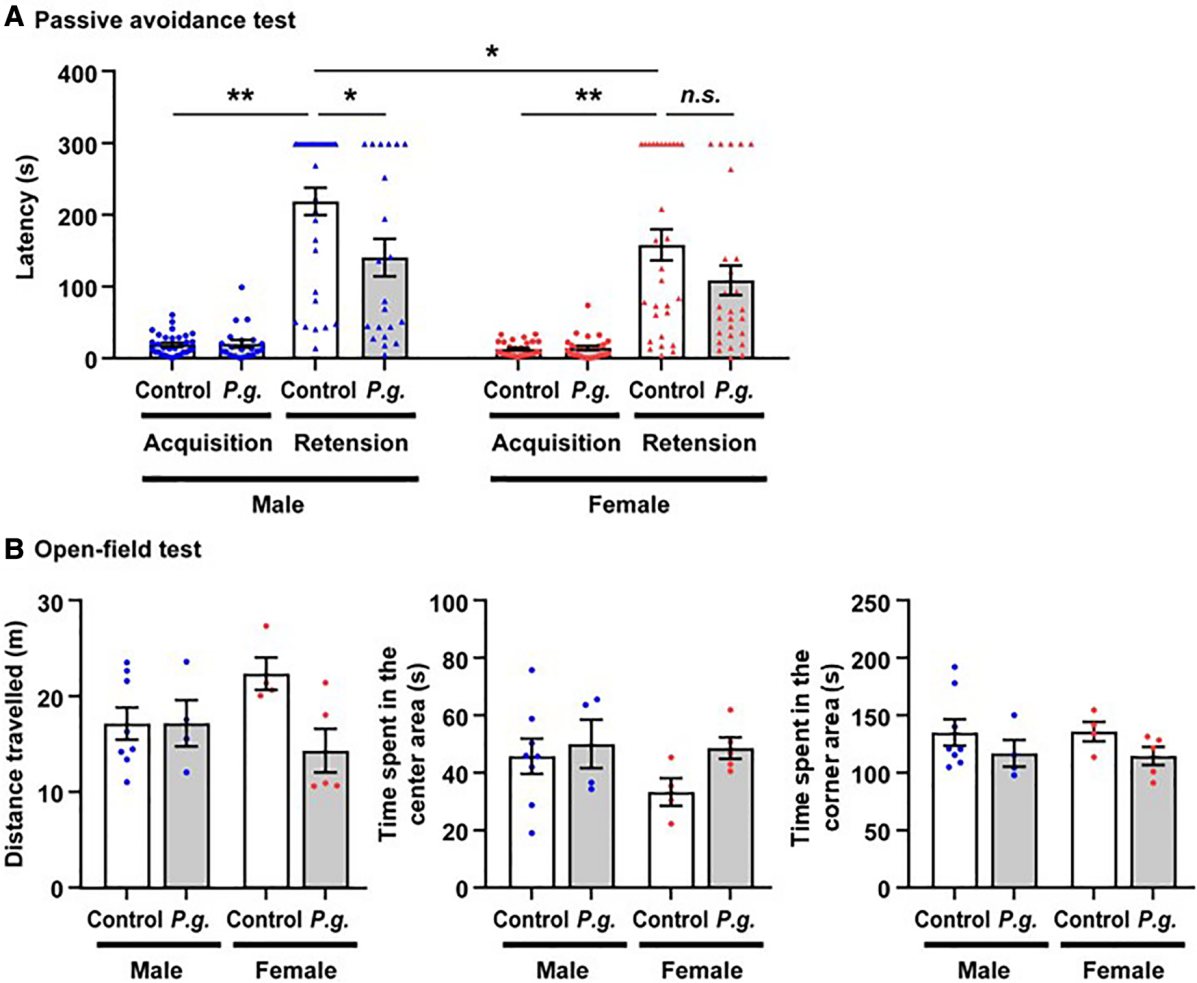
The mean latency of step-through in the passive avoidance test of mice offspring. (**A**) Data are shown as the mean ± SE. *, *p* < 0.05; **, *p* < 0.01. controls: *n* = 62 (males, *n* = 32; females, *n *= 30); *P. gingivalis*: *n* = 48 (males, *n* = 21; females, *n* = 27). (**B**) the mean times spent in the central and corner area, and the total distances traveled between *P. gingivalis* group and control group by open-field test. Data are shown as the mean ± SE. controls: *n* = 12 (males, *n* = 8; females, *n *= 4); *P. gingivalis*: *n* = 9 (males, *n* = 4; females, *n* = 5).

There was no significant difference in the latency of the acquisition trial on Day 1 between the *P. gingivalis* and control groups in males (21.9 ± 5.1 vs. 20.8 ± 2.6 s) or females (16.2 ± 3.0 vs. 14.3 ± 1.8 s). In both the *P. gingivalis* and control groups, the latency of the retention trial on Day 2 was significantly longer than that of the acquisition trial in males (141.9 ± 25.9 and 220.0 ± 18.9 s, respectively) and females (110.0 ± 20.6 and 159.6 ± 21.7 s, respectively). The latency of the retention trial in the control group was significantly longer in males than in females. Additionally, the latency of the retention trial was significantly shorter in the *P. gingivalis* group than in the control group in males (*p* < 0.05; Figure 2A). A repeated measures ANOVA revealed the significant main effects of *P. gingivalis* infection (*F*_1,106_ = 7.716, *p* < 0.01), sex (*F*_1,106_ = 5.401, *p* < 0.05), acquisition/retention trial (*F*_1,106_ = 170.074, *p* < 0.0001), and interaction between *P. gingivalis* infection and acquisition/retention trial (*F*_1,106_ = 9.321, *p* < 0.01). In the open-field test, there was no difference in the distance travelled, time spent in the center area, or time spent in the corner area between the *P. gingivalis* and control groups in either males or females (Figure 2B). These results suggest that the learning and memory abilities of the *P. gingivalis* group were lower than those of the control group. Since there was no significant interaction between *P. gingivalis* infection and sex in the passive avoidance test (*F*_1,106_ = 0.425, *p* > 0.05) or open-field test (*F*_1,17_ = 3.671, *p* > 0.05 for distance travelled; *F*_1,17_ = 0.707, *p* > 0.05 for time spent in the center area; *F*_1,17_ = 0.017, *p* > 0.05 for time spent in the corner area), subsequent analyses were performed without distinguishing between males and females.

### 3.2. Immunohistochemical evaluation of the hippocampus

#### 3.2.1. *P. gingivalis* inoculated from the maternal oral cavity was detected in the brains of offspring

**Figure 3:**
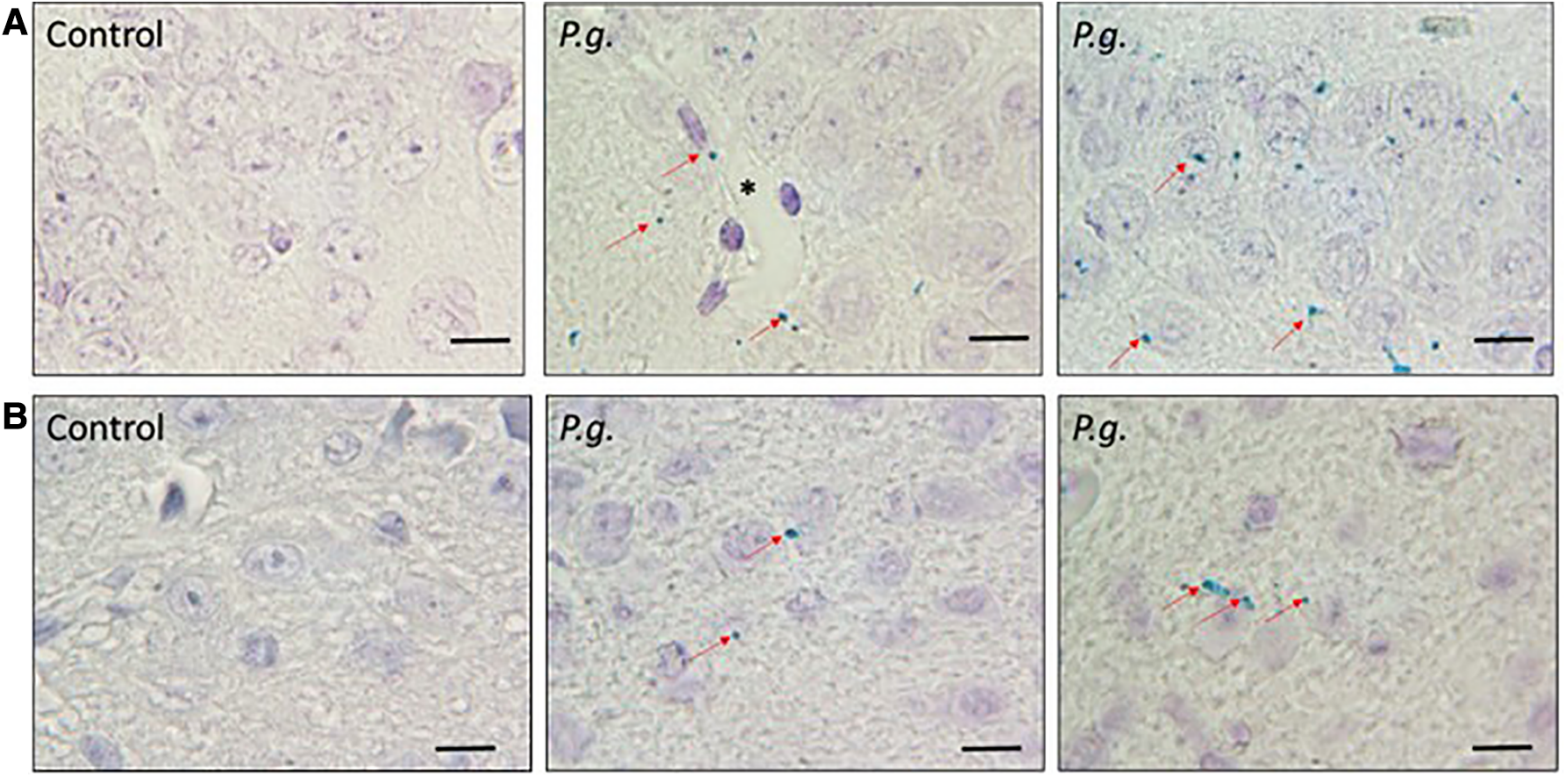
Immunohistochemical analysis of *P. gingivalis*. *P. gingivalis* immunostaining (green) in (**A**) the hippocampus and (**B**) the amygdala in the *P. gingivalis* group. Scale bar = 10 µm. Red arrows = *P. gingivalis*. *, Blood vessel.

*P. gingivalis* was sporadically observed in the hippocampus in the *P. gingivalis* group (Figure 3A), especially in and around neurons and vascular endothelial cells. *P. gingivalis* was also observed in the amygdala, but to a lesser extent (Figure 3B).

#### 3.2.2. Maternal *P. gingivalis* infection induced hippocampal neurodegeneration in offspring

**Figure 4:**
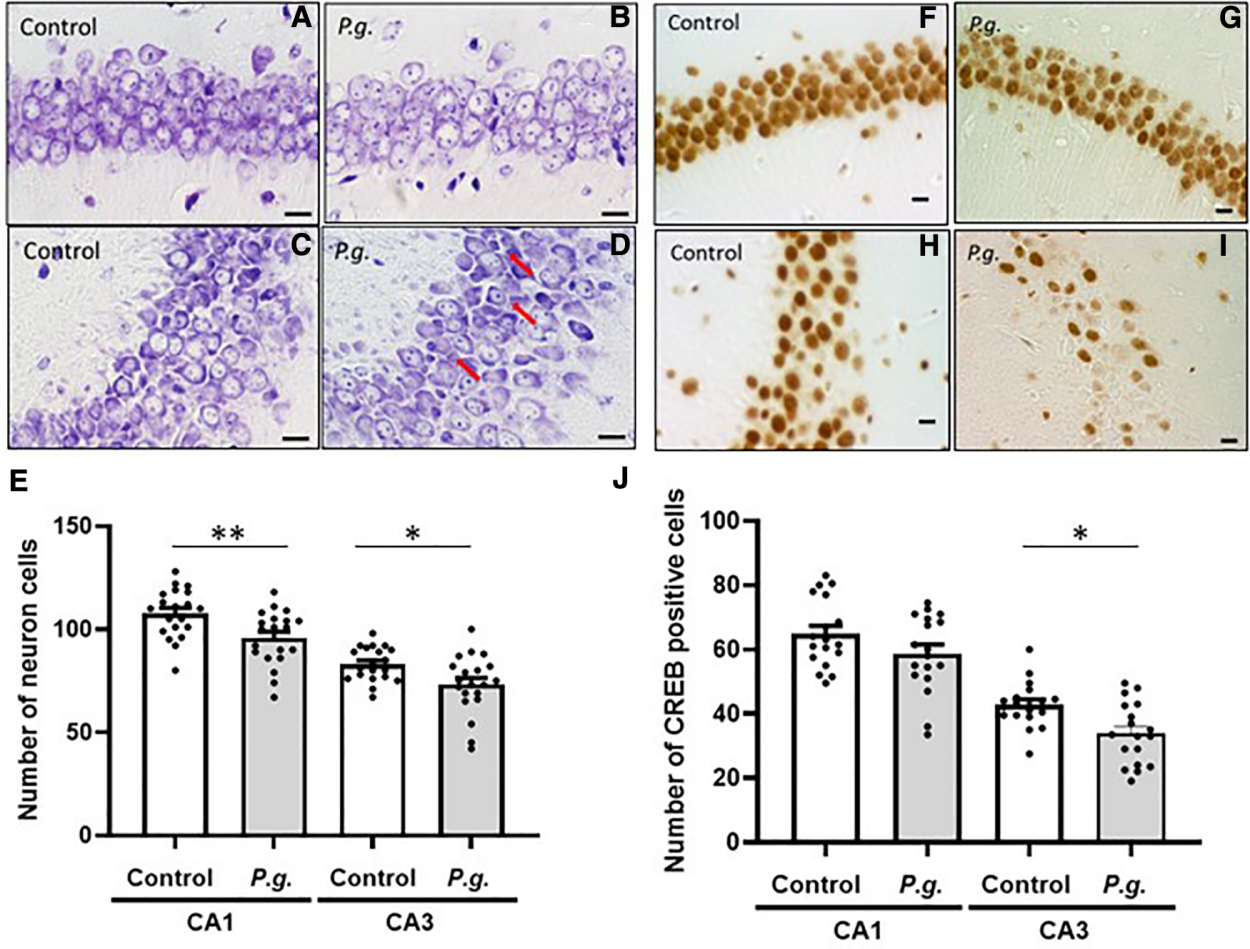
Effects of maternal *P. gingivalis* infection on neurons in the hippocampus of offspring. Nissl staining in (**A**,**B**) CA1 and (**C**,**D**) CA3. (**E**) Number of neurons in CA1 and CA3(*n *= 20). CREB immunostaining in (**F**,**G**) CA1 and (**H**,**I**) CA3. (**J**) Number of CREB-positive cells in CA1 and CA3(*n *= 18). Data are shown as the mean ± SE. *, *p *< 0.05; **, *p* < 0.01. Scale bars = 10 µm. CREB, cAMP response element binding protein.

Microscopic examination of Nissl-stained sections revealed a decrease in the number of CA1 and CA3 neurons in the *P. gingivalis* group (Figures 4A–D). Many degenerated neurons were observed in the CA3 region (Figure 4D). The average number of neurons in the CA1 and CA3 regions were 95.9 ± 2.9 and 107.8 ± 2.7, respectively, in the *P. gingivalis* group and were 73.3 ± 3.2 and 83.1 ± 1.9, respectively, in the control group. The number of neurons in the CA1 and CA3 regions was significantly lower in the *P. gingivalis* group than in the control group (*p* < 0.01 and *p* < 0.05, respectively; Figure 4E).

#### 3.2.3. Maternal *P. gingivalis* infection reduced hippocampal CREB expression in offspring

The expression of CREB in the CA1 and CA3 regions of the hippocampus is shown in Figures 4F–I. The number of CREB-positive cells in the *P. gingivalis* group tended to decrease in the CA1 region and was significantly decreased in the CA3 region (*p* < 0.01; Figure 4J).

#### 3.2.4. Maternal *P. gingivalis* infection activated hippocampal microglia and astrocytes in offspring

**Figure 5:**
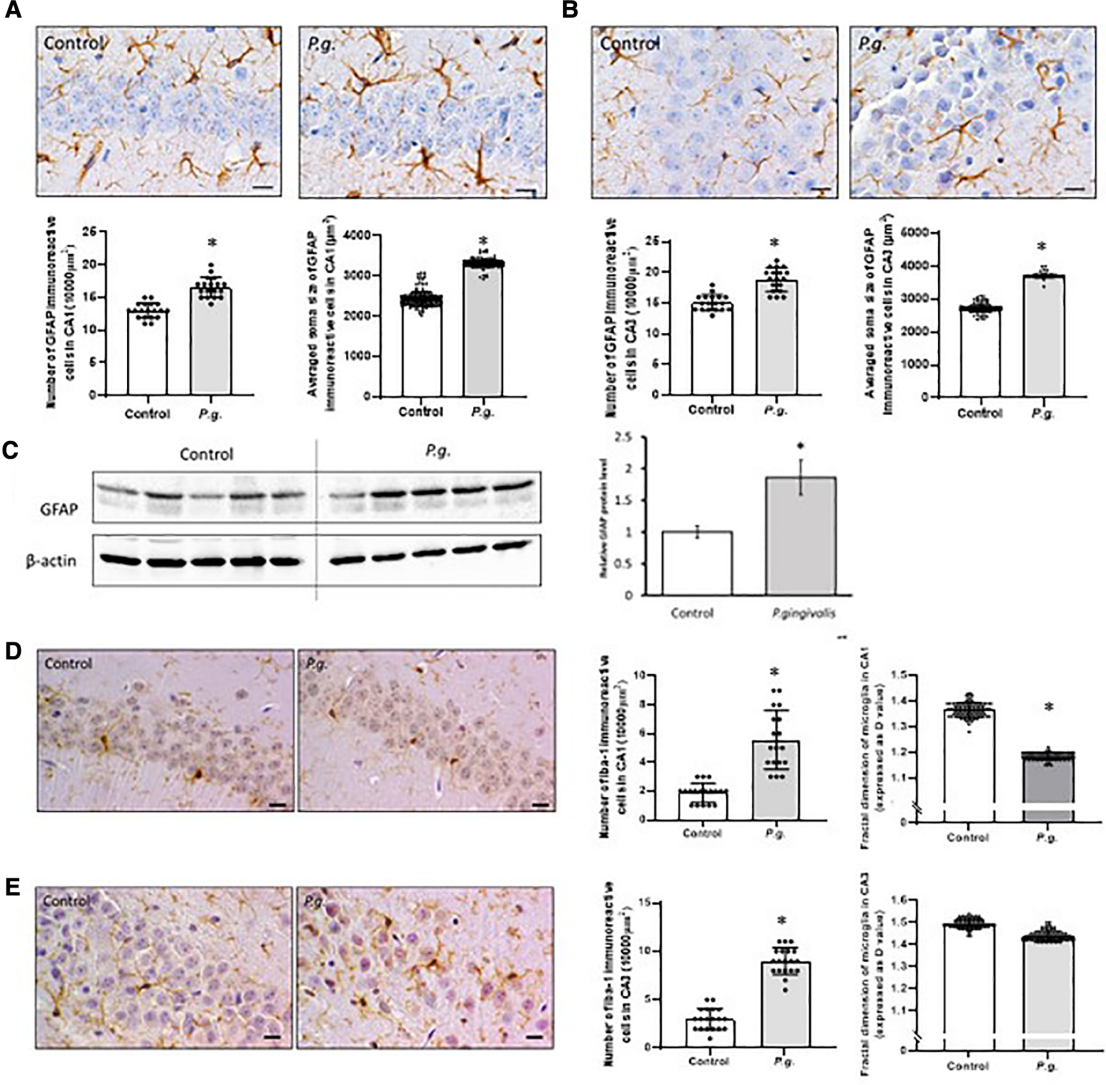
Effects of maternal *P. gingivalis* infection on astrocytes and microglia in the hippocampus of offspring. GFAP immunostaining in (**A**) CA1 and (**B**) CA3(*n *= 10). (**C**) GFAP protein expression by Western blotting (*n *= 5). Iba1 immunostaining in (**D**) CA1 and € CA3(*n *= 10). Data are shown as the mean ± SE. *, *p* < 0.05. Scale bars = 10 µm. GFAP, glial fibrillary acidic protein; Iba1, ionized calcium binding adapter protein 1.

In this study, the number of GFAP-positive astrocytes with enlarged soma in the hippocampus was increased in the *P. gingivalis* group. Numerous protrusions of GFAP-positive astrocytes were observed, especially around damaged neurons. Both the number and mean soma size of GFAP-positive astrocytes in the CA1 and CA3 regions were significantly increased in the *P. gingivalis* group (Figures 5A,B). Western blotting showed that hippocampal GFAP expression was approximately 2-fold higher in the *P. gingivalis* group than in the control group (*P* < 0.05; Figure 5C).

The number of Iba1-positive cells associated with neuroinflammation in the CA1 and CA3 regions was significantly higher in the *P. gingivalis* group than in the control group. In the *P. gingivalis* group, Iba1-positive microglia had oval soma with short projections and macrophage-like morphology. Microglial morphology was analyzed using “fractal dimension.” D values generally decrease as cells cycle toward a more activated state and then increase as they return to a ramified state (31). The D values of microglia in the amygdala were decreased significantly in the *P. gingivalis* group (*p* < 0.05; Figures 4D,E).

### 3.3. Immunohistochemical evaluation of the amygdala

#### 3.3.1. Maternal *P. gingivalis* infection reduced the number of neurons and CREB-positive cells in the amygdala of offspring

**Figure 6:**
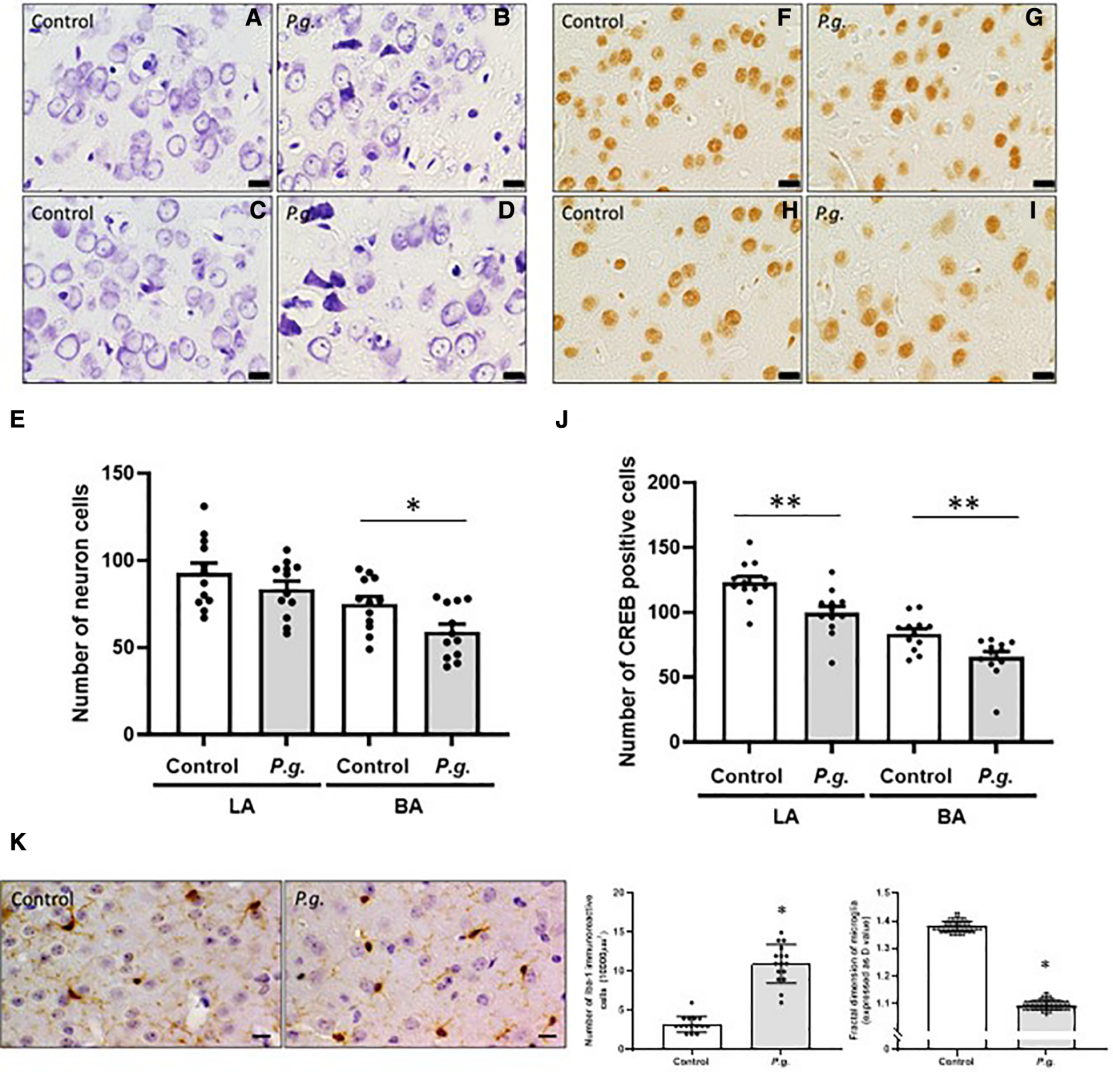
Effects of maternal *P. gingivalis* infection on microglia in the amygdala of offspring. Nissl staining in (**A**,**B**) the lateral and (**C**,**D**) the basal amygdala. (**E**) Number of neurons (*n *= 12). CREB immunostaining in (**F**,**G**) the lateral and (**H**,**I**) the basal amygdala. (**J**) Number of CREB-positive cells (*n *= 12). (**K**) Iba1 immunostaining in the basal amygdala (*n *= 10). Data are shown as the mean ± SE. *, *p* < 0.05; **, *p *< 0.01. Scale bar = 10 µm. CREB, cAMP response element binding protein; Iba1, ionized calcium binding adapter protein 1.

The amygdala is important for processing emotions and memories associated with fear. The basolateral complex consists of basal and lateral nuclei, which play a crucial role in perceiving and responding to fear (32, 33). We subsequently examined the histological changes in the basal and lateral amygdala. The number of degenerated neurons increased in both the basal and lateral amygdala in the *P. gingivalis* group (Figures 6A–D). The number of neurons in the basal and lateral amygdala was 59.1 ± 4.4 and 75.0 ± 4.3, respectively, in the *P. gingivalis* and was 83.6 ± 4.6 and 92.8 ± 5.8, respectively, in the control group. In the *P. gingivalis* group, the number of neurons in the lateral amygdala tended to decrease, whereas the number of neurons in the basal amygdala significantly decreased (*p* < 0.05; Figure 6E). The number of CREB-positive cells in both the basal and lateral amygdala were significantly lower in the *P. gingivalis* group than in the control group (*p* < 0.01; Figures 6F–J).

#### 3.3.2. Maternal *P. gingivalis* infection induced neuroinflammation in the amygdala of offspring

The number of Iba1-positive cells was significantly higher in the amygdala in the *P. gingivalis* group than in the control group (*p* < 0.05). The D values of microglia decreased significantly in the amygdala in the *P. gingivalis* group (*p* < 0.05; Figure 5K), indicating morphologically activated microglia.

## 4. Discussion

Using a pregnant mouse model, this study demonstrated that *P. gingivalis* inoculated from the maternal oral cavity was transferred to the brain of the offspring. Behavioral tests showed that the offspring of *P. gingivalis*-infected mothers had cognitive impairment, with reduced numbers of neurons and CREB-positive cells and increased numbers of activated microglia and reactive astrocytes, in the hippocampus. In the amygdala, there was prominent dysfunction caused by reduced numbers of neurons and CREB-positive cells and microglial activation.

Our behavior tests indicated that latency time in *P. gingivalis* group is significantly shorter than that in control group by passive avoidance test, but no difference in time spent in center and corner area, and total distances traveled by open-field test to evaluate spontaneous motor/exploratory activity in mice. It is suggested that maternal odontogenic infection of *P. gingivalis* may induce cognitive dysfunction, but not affect spontaneous motor/exploratory activity of offspring.

Intrauterine infection with various bacteria/viruses leads to neurological disorders in children. Maternal infection with influenza virus and rubella and cytomegalovirus increases the risk of schizophrenia (34) and autism spectrum disorder (35, 36), respectively, in the offspring. Intraperitoneal injection of pregnant mice with *Escherichia coli* lipopolysaccharide causes long-term changes in the offspring, including increased anxiety-related behaviors and longer time required to adapt to social situations (37). A model of local inflammation induced by intrauterine administration of *E. coli* lipopolysaccharide also showed reduced cognitive function in children (17).

Chávez et al. (38) recently reported that maternal exposure to lipopolysaccharide alters the fetal brain microenvironment and enhances hemichannel and pannexon activity, which is associated with astrocyte signaling and gliotransmitter release. Enhanced hemichannel and pannexon activity may persist for a lifetime and increase sensitivity to substances that cause neuronal injury, such as lipopolysaccharide. Lipopolysaccharide-induced inflammation during fetal development leads to excessive proinflammatory cytokine production upon lipopolysaccharide administration, especially in adult males (16). It is possible that *P. gingivalis*, a major periodontal pathogen that also has lipopolysaccharide, enters the bloodstream, and induces fundamental structural/functional alterations in the fetal brain as a first hit. These offspring may then be more susceptible to behavioral abnormalities and neurological disorders after a second hit later in life.

An *in vitro* study (39) showed that *P. gingivalis* infects neurons and induces apoptosis. In this study, we observed a reduction in the number of neurons in the CA1 and CA3 regions of the hippocampus and the amygdala, suggesting that *P. gingivalis* penetrates the blood–brain barrier (BBB), leading to a decline in brain function in the offspring. However, further TUNEL and ssDNA staining to confirm apoptosis in neurons did not detect apoptosis. This could be explained by the fact that the results of this study were collected at only one time point (i.e., 45 days after birth), and thus, it is thought that it was difficult to detect apoptosis. Several time points after birth should be observed to examine the mechanism. It was possible that the neurons were decreased as *P. gingivalis* infected the neurons and caused apoptosis or that the number of neurons was originally low during development in utero. Further investigation is needed.

The *P. gingivalis* group also showed a reduced number of CREB-positive cells in the CA1 and CA3 regions of the hippocampus and the basal and lateral amygdala. CREB is an important transcription factor for memory formation. Previous studies suggested that CREB does not affect learning or short-term memory, but it is important for long-term memory (40, 41). Prolonged latency in the passive avoidance test is caused by the recognition and consolidation of the memory of a fearful experience of electrical stimulation in the acquisition trial. The hippocampus is important for memory formation, and the amygdala is responsible for the recognition of fearful experiences. CREB activation in the amygdala has been shown to affect fear-dependent learning (40, 42, 43). A *P. gingivalis* infection-induced reduction in the numbers of neurons and CREB-positive cells may have resulted in reduced latency in the passive avoidance test.

Neuroinflammation triggers behavioral alterations and cognitive impairments in many neurological disorders. Microglia play an important role in the development of innate immunity and neuroinflammation in the brain. Under normal conditions, microglia are in a quiescent state with small cell bodies and elongated processes with fine branching. Under pathological conditions, such as infection or invasion, microglia become activated with enlarged cell bodies and short processes (44, 45). Studies in an adult mouse model have shown that *P. gingivalis* and *P. gingivalis* lipopolysaccharide activate microglia in the hippocampus and cortex (46, 47). In addition, lipopolysaccharide-induced neuroinflammation has been shown to activate microglia in the amygdala (48). However, lipopolysaccharide has also been shown to induce glial activation in the hippocampus but not in the amygdala (49). Intrauterine exposure to lipopolysaccharide induces microglial activation in the amygdala in the pre- and postnatal brain of the offspring (50). Thus, the effect of neuroinflammation on functional changes in the amygdala is still unclear. Our results showed that *P. gingivalis* induced neuroinflammation in the hippocampal–amygdala circuit in the *P. gingivalis* group, with increased microglial activation.

Astrocytes become reactive in response to central nervous system injury, including infection, trauma, ischemia, and neurodegenerative diseases, with changes in molecular expression and morphology characterized by hypertrophy and increased GFAP expression (mesenchymal filaments). The scar formation process, known as astrogliosis, acts as a repair mechanism against neuronal injury (51). Reactive astrocytes release proinflammatory cytokines that cause neuropathic pain (52). In this study, we observed changes in astrocyte morphology and increased GFAP expression, suggesting astrocyte activation. However, it was unclear whether reactive astrocytes had neuropathic or restorative effects.

*P. gingivalis* was detected in the brains of wild-type mice after discontinuing oral *P. gingivalis* administration in a previous study (21). In the current study, *P. gingivalis* continued to persist in the brain, resulting in fewer neurons in the hippocampus. Therefore, we considered that the behavior and histological changes observed in the brain of offspring at 45 days of age may have resulted from neurodegeneration/neuroinflammation caused by *P. gingivalis* infection in the brain. However, the fetus is exposed to *P. gingivalis* infection during development. The possibility that abnormalities in neuronal development may have caused neuronal loss and functional deficits cannot be ruled out, and further studies are needed.

In this study, *P. gingivalis* was diffusely distributed throughout the brain, especially in the hippocampus. *P. gingivalis* has been shown to translocate to the brain in APOE^–/–^ mice with oral *P. gingivalis* infection, in which *P. gingivalis* is detected in hippocampal neurons and around blood vessels (53, 54). *P. gingivalis* has also been detected in the nucleus and periphery of hippocampal neurons, astrocytes, and microglia in studies of wild-type mice exposed to oral *P. gingivalis* administration (46). The current study also found *P. gingivalis* localized in and around vascular endothelial cells in the brain, suggesting that *P. gingivalis* enters the brain via the BBB. Previously we reported immunolocalization of *P. gingivalis* in capillaries on the fetal side of *P. gingivalis*-infected placentas (55, 56), suggesting that *P. gingivalis* enters the fetal bloodstream via the placenta. For maternal *P. gingivalis* to translocate to the fetal brain, it must cross two barriers: the placental blood barrier, which prevents the passage of foreign substances from the mother to the fetus, and the BBB, which prevents the passage of foreign substances into the brain.

*P. gingivalis* increases vascular permeability by disrupting the endothelial barrier and intercellular junctions, suggesting that *P. gingivalis* enters the brain via the BBB (57). In a mouse model, *P. gingivalis* infected aortic endothelial cells, induced endothelial dysfunction and apoptosis, and invaded the smooth muscle layer of the aortic wall (58). *P. gingivalis* may also increase TNF-α and IL-1b expression, induce cell death, and increase the permeability of the BBB to invade brain endothelial cells (59). Gingipain, a virulence factor of *P. gingivalis*, also causes BBB dysfunction by degrading tight junction proteins (60). This requires further investigation. The placental blood barrier and BBB are not the only translocation routes. Previous studies have reported that *P. gingivalis* may enter the brain via the bloodstream (61, 62) by penetrating the periventricular organ and perivascular space (22) or by translocating along the olfactory and trigeminal nerves (63).

Maternal–fetal blood circulation in the placenta is not the only route of transmission to the infant. *P. gingivalis* has also been detected in amniotic fluid (14). Therefore, it is possible that *P. gingivalis* may be ingested through amniotic fluid. Although there have been no reports of *P. gingivalis* infection from breast milk, it is possible that *P. gingivalis* may be transmitted through breast milk in the same way as cytomegalovirus (64), human immunodeficiency virus (65), and group B streptococcus (66). Further studies are needed to determine the route of invasion of *P. gingivalis* into the brain of the offspring.

We cannot rule out the possibility that the mice were stressed by *P. gingivalis* odontogenic infection. Stress such as pain in the oral area results in enhanced nociception (67). However, in a rat model of chronic apical periodontitis, body weight decreased temporarily but did not change over time, and stress markers were not increased (68). We previously reported that *P. gingivalis* odontogenic infection induced PTB, using the same mouse model in which *P. gingivalis* was directly inoculated in the dental pulp. In this mouse model, periapical granuloma was established as a reservoir of *P. gingivalis* 6 weeks after *P. gingivalis* inoculation (at mating). It is well accepted that periapical granuloma is usually asymptomatic and remain quiescent for long periods. We also reported that compared with control mice, mice with *P. gingivalis* odontogenic infection did not show abnormal behaviors and body weight changes over 6 weeks (19), indicating that *P. gingivalis* odontogenic infection could not induce marked stress such as pain during the pregnancy period.

*P. gingivalis* has been found to be involved in cognitive impairment, such as AD (6, 22, 69, 70); LPS and gingipain, pathogens of *P. gingivavlis,* as well as *P. gingivalis* bacteria themselves, have been detected in the hippocampus and cerebral cortex of AD patients (21, 22). *P. gingivalis* were also detected in the hippocampus and cortex in this study. However, it is still unclear the mechanisms that the *P. gingivalis* themselves and their pathogenic factors work in the brain. Recently, Li et al. reported that in the hippocampus of the mice with *P. gingivalis*-LPS, the expression of TNF-α and IL-1β significantly increased through astrocyte and microglia- activation, resulting in depression-like behaviors (71). It is also reported that *P. gingivalis* and/or its vesicle applied into gingival sulcus of mice induced memory impairment-like behaviors through upregulation of TNF-α expression and microglial activation in hippocampus tissue (72).

Moreover, these cytokines like TNF-α and IL-1β, which are produced from local periodontitis area, may reach the brain, and promote the AD pathology (69, 71). *P. gingivalis*-LPS increased serum IL-1β and decreased tight junction protein, induce depression-like behavior by initiating the neuroinflammation through damaging the permeability of BBB (72). In addition, it has also been reported that IL-6 in serum induces neuroinflammation in the hippocampus and destruction of the BBB in a mouse model with experimental periodontitis (73). Since in the present study, *P. gingivalis* has been detected in the brains of offspring, it has possibility that the virulence factor of *P. gingivalis* is also transferred.

Thus, it is gradually evident that the oral microbiome-host interactions plays an important role in development of various behavior abnormalities AD (73).

In addition, inflammatory cytokines produced in maternal body are also likely to be transferred to the fetus but were not detected significant differences in serum cytokines-concentration of 45-day-old offspring in this experiment (the figure was not shown). Our previous studies have shown that Gal-3 (an immune regulatory molecule), which can induce immune mediators (such as TNF-α and COX-2) in the placenta, is involved in mechanism of preterm birth caused by *P. gingivalis*-odontogenic infection. So, we can examine the effects of suppressing inflammatory cytokines in the maternal body by administering Gal-3 inhibitors, which can also be safely administered to pregnant mice, on offspring's brain tissue and function. We consider the experiment as future research.

A limitation for this study is that only animal experiments were used to get conclusion. It is true that animal studies do not completely predict human outcomes. Therefore, a future prospective clinical study of children born from mothers with periodontitis is needed to clarify the relationship between maternal periodontitis and offspring's cognitive dysfunction. Another limitation is that the number of animals used in open field test to show spontaneous motor and exploratory is still small. Additional open field experiment using more animals are necessary to clarify the effect of maternal periodontitis on motor/exploratory action, anxiety and or willingness of offspring.

In conclusion, the animal study indicated that the offspring of *P. gingivalis*-infected mother mice have behavioral abnormalities. The underlining mechanisms may involve neurodegeneration/neuroinflammation in the hippocampus and amygdala caused by *P. gingivalis* infection in the brain.

## Data Availability

The raw data supporting the conclusions of this article will be made available by the authors, without undue reservation.

## Supplementary material

Supplementary Figure 1 The number of offspring per litter used (A). Body weight at 45 days of age, in which behavior tests were applied (B). male (left), female (right).
