# Supplementary figures and images for: Mouse maternal odontogenic infection with Porphyromonas gingivalis induces cognitive decline in offspring
Source: Front Pediatr. 2023 Aug 11;11:1203894. doi: 10.3389/fped.2023.1203894 (PMC10450928; doi:10.3389/fped.2023.1203894)

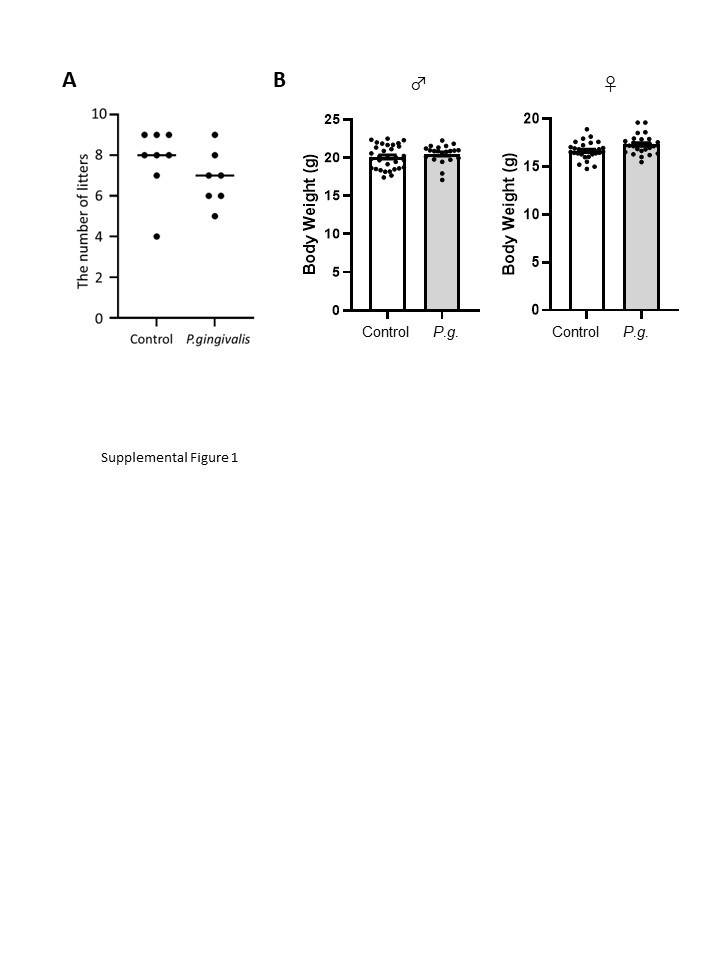

Supplement: Supplementary Figure 1 — The number of offspring per litter used (A). Body weight at 45 days of age, in which behavior tests were applied (B). male (left), female (right). [file Image1.jpeg]
